# Comment on Kehrer et al. Using High-Resolution Ultrasound to Assess Post-Facial Paralysis Synkinesis—Machine Settings and Technical Aspects for Facial Surgeons. *Diagnostics* 2022, *12*, 1650

**DOI:** 10.3390/diagnostics12102431

**Published:** 2022-10-08

**Authors:** Charles Nduka, Ruben Yap Kannan, Gerd Fabian Volk, Orlando Guntinas-Lichius

**Affiliations:** 1 Facial Palsy Unit, Department of Plastic & Reconstructive Surgery, Queen Victoria Hospital, East Grinstead RH19 3DZ, UK; 2 Facial-Nerve-Center Jena, Department of Otorhinolaryngology, Center of Rare Diseases Jena, Jena University Hospital, Am Klinikum 1, 07747 Jena, Germany

In “Using High-Resolution Ultrasound to Assess Post-Facial Paralysis Synkinesis—Machine Settings and Technical Aspects for Facial Surgeons”, Andreas Kehrer et al. present ultrasound (US) device settings for facial muscle examination to be used by facial surgeons to improve their workflow and enhance their image quality [1]. The step-by-step structured working protocol, starting with basic but very important machine settings, should be helpful to conduct more insightful examinations in patients with different kinds of facial palsies.

We strongly encourage the use of facial US not only to capture high-quality US images, but also to quantify these images to acquire metrics related to the various muscles. Indeed, we have previously published detailed Instructions for Sonography of the Mimic Musculature as a part of the book Management of Post-Facial Paralysis Synkinesis [2].

These instructions for sonography of the mimic musculature help sonographers to better understand the complex sonographic cross-sections of the mimic and masticatory musculature and help one to achieve reproducible and quantifiable scans. Because of the special anatomy of the mimic musculature, it is not always easy to differentiate single mimic muscles from the surrounding fat and connective tissue. Therefore, all sonographic images in the aforementioned guide are accompanied by a schematic drawing to help make things clearer. In these instructions to clarify the dynamic changes of the different muscles in motion, each sonographic image in relaxation is accompanied by an image in maximum arbitrary contraction. The anatomic structures marked with numbers are named and explained underneath the appropriate images.

A similar detailed instruction with ultrasound and schematic pictures was published by Wu in 2022, discussing potential clinical relevance for every muscle [3].

Therefore, for a surgeon starting US of the face, reading both publications should be useful to acquire high-quality quantitative data for scientific research and evidence-based medicine [4,5,6,7,8].

Anyway, similar to photos or videos, ultrasound for assessing facial function and deficits can only quantify some aspects. Therefore, the gold standard should be a multimodal assessment using not only imaging and/or electrophysiological techniques, but also patient and expert related outcome measures and ideally (but nearly never used) ratings of lay persons.
